# Pore Ordering in Anodic Aluminum Oxide: Interplay between the Pattern of Pore Nuclei and the Crystallographic Orientation of Aluminum

**DOI:** 10.3390/nano12091417

**Published:** 2022-04-20

**Authors:** Ilya V. Roslyakov, Stepan V. Sotnichuk, Sergey E. Kushnir, Lev A. Trusov, Ivan V. Bozhev, Kirill S. Napolskii

**Affiliations:** 1 Department of Materials Science, Lomonosov Moscow State University, 119991 Moscow, Russia; ilya.roslyakov@gmail.com (I.V.R.); sotnya777@mail.ru (S.V.S.); kushnir@elch.chem.msu.ru (S.E.K.); 2 Kurnakov Institute of General and Inorganic Chemistry RAS, 119991 Moscow, Russia; 3 Dukhov Research Institute of Automatics (VNIIA), 127055 Moscow, Russia; 4 Department of Chemistry, Lomonosov Moscow State University, 119991 Moscow, Russia; trusov@inorg.chem.msu.ru; 5 Department of Materials Science, MSU-BIT University, Shenzhen 517182, China; 6 MSU Quantum Technology Centre, 119991 Moscow, Russia; bozhjev.ivan@physics.msu.ru; 7 Department of Physics, Lomonosov Moscow State University, 119991 Moscow, Russia

**Keywords:** anodic aluminum oxide, focused ion beam, single crystal substrate, defect-free array, crystallographic orientation

## Abstract

Anodization of aluminum with a pre-patterned surface is a promising approach for preparing anodic aluminum oxide (AAO) films with defect-free pore arrangement. Although pronounced effects of crystallographic orientation of Al on the AAO structure have been demonstrated, all current studies on the anodization of pre-patterned aluminum consider the substrate as an isotropic medium and, thus, do not consider the azimuthal orientation of the pattern relative to the basis vectors of the Al unit cell. Here, we investigate the interplay between the azimuthal alignment of the pore nuclei array and the crystallographic orientation of aluminum. Al(100) and Al(111) single-crystal substrates were pre-patterned by a Ga focused ion beam and then anodized under self-ordering conditions. The thickness-dependent degree of pore ordering in AAO was quantified using statistical analysis of scanning electron microscopy images. The observed trends demonstrate that the preferred azimuthal orientation of pore nuclei rows coincides with the <110> directions in the Al unit cell, which is favorable for creating AAO with a high degree of pore ordering. In the case of an unspecified azimuthal orientation of the pore nuclei array, crystallography-affected disorder within the AAO structure occurs with increasing film thickness. Our findings have important implications for preparing defect-free porous films over 100 µm in thickness that are crucial for a variety of AAO applications, e.g., creating metamaterials and 2D/3D photonic crystals.

## 1. Introduction

Since the beginning of the 20th century, aluminum anodization has been applied industrially for surface finishing, corrosion protection, and preparing dielectric films for electrolytic capacitors [1,2,3]. Electrolytes with pH 5–7, in which the anodic aluminum oxide (AAO) is insoluble, are used for the formation of barrier (non-porous) alumina films [4,5]. The anodization in acidic electrolytes (e.g., solution of sulfuric and oxalic acids) results in porous AAO, which consists of cylindrical channels grown preferably along the surface normal. The in-plane arrangement of pores, as well as the interpore distance (*D*_int_) and pore diameter (*D*_p_), can be tuned by changing the anodizing conditions, e.g., electrolyte composition [6,7,8], anodizing voltage [9,10,11], temperature [12,13,14], and inter-electrode distance [15]. Quantitative determination of the degree of pore ordering in AAO structure is performed on the basis of statistical analysis [16] or fast Fourier transform [17,18] of scanning electron microscopy (SEM) images, and by small-angle scattering of X-rays [19] or neutrons [20]. It has been shown that self-ordering of AAO structure occurs under potentiostatic anodization on the upper voltage limit of the kinetic control region and in the diffusion control regime [10,21]. The in-plane pore ordering accompanied by the formation of domains, i.e., defect-free micron-sized areas with a hexagonal arrangement of pores. Long-term anodization can enhance the domain size [22,23,24]. Furthermore, coarse-grained aluminum foils with texture preferences [20,25] or single crystals with a distinct crystallographic orientation [26,27] are recommended for use as substrates to obtain AAO with an ordered structure.

Another approach for preparing well-ordered porous structures includes the anodization of aluminum with a pre-patterned surface [28]. To create a defect-free hexagonal array of concaves on the Al surface, nanoimprinting [29,30], electron beam and laser interference lithography [31,32], and focus ion beam (FIB) etching [33,34] approaches are used. In the subsequent anodization process, these concaves act as nucleation centers. As a rule, the anodizing of pre-patterned aluminum should be conducted under a kinetic regime at an appropriate anodizing voltage (*U*), providing an interpore distance equal to the concave-to-concave distance; otherwise, defect-free AAO structures inherited from the patterned concave array can disorder even at AAO film thicknesses of as low as ca. 10 µm [35]. At higher AAO thicknesses, diffusion limitations [36] or incorrect azimuthal orientation of an array of concaves relative to the Al unit cell [37] may destroy the hexagonal arrangement of pores.

The impacts of aluminum’s crystallographic orientation on the porous structure of AAO can be summarized in the following statements. (i) The average azimuthal orientation of the pore domains [37] and the out-of-plane pore growth direction [38] retain the aluminum microstructure: these remain constant within each grain, whereas a transition through a grain boundary leads to a sudden change in these parameters. (ii) Anodization of Al(100) results in the fewest point defects in the porous structure [26,39] and the lowest pore tortuosity [40]. (iii) Anodization of Al(111) leads to the smallest mosaicity of the porous structure (i.e., spread of domains’ azimuthal orientations) [27] and the slowest linear growth of the average domain size with anodizing duration, which is limited solely by the size of the single-crystal substrate [41]. (iv) Al(110) substrates should be avoided in the preparation of AAO due to the formation of numerous point defects and high pore tortuosity [26,39]. Despite such pronounced effects of crystallographic orientation, all up-to-date studies of the anodization of pre-patterned aluminum consider the substrate as an isotropic medium.

Here, the impact of aluminum crystallographic orientation on the ordering of the porous structure during the anodization of Al(100) and Al(111) single crystals pre-patterned by FIB is studied for the first time. The thickness evolution of the AAO porous structure with varying azimuthal orientations relative to the Al unit cell is determined using SEM and subsequent statistical analysis of the SEM images. A quantitative comparison of the degree of pore ordering in AAO grown on the pre-patterned Al single crystals with that of AAO formed on substrates without patterning is also performed.

## 2. Materials and Methods

Al(100) and Al(111) single crystals (MTI Corporation, Richmond, CA, USA) with a purity of 99.99%, orientation accuracy of 2°, and size of 10 × 10 × 1 mm^3^ were used as substrates. Before anodization, single crystals were electrochemically polished in a solution containing 1.8 M CrO_3_ and 12.9 M H_3_PO_4_ at 80 °C. Forty pulses of anodic polarization at a current density of 0.4 A cm^−2^ with a duration of 3 s and a pulse-to-pulse interval of 40 s were imposed.

A recently proposed approach was applied in this work to prevent Ga incorporation into the Al surface layer during Ga FIB patterning [42]. In summary, the Al surface was covered by a protective barrier-type alumina layer using a linear voltage sweep up to 40 V (sweep rate of 0.5 V s^−1^) in 0.1 M H_3_PO_4_ at 1 °C (first anodizing stage). Next, the crystallographic orientation of the aluminum was examined using the electron backscatter diffraction (EBSD) technique. The single-crystal substrate was then rotated in-plane until the target azimuthal orientation of the Al unit cell was established. Subsequent patterning using Ga FIB resulted in a 50 × 40 µm^2^ array of concaves arranged hexagonally with a concave-to-concave distance of 106 nm. The second anodizing stage was carried out in the same manner as the first stage. Selective etching of the barrier-type alumina layer in a solution containing 0.2 M CrO_3_ and 0.6 M H_3_PO_4_ at 70 °C was then performed to obtain an array of round-shaped concaves on the Al surface.

Pre-patterned single crystals were anodized in a two-electrode electrochemical cell. The Al substrate was pressed to the bottom of the cell with an O-ring seal (internal diameter of 0.6 cm) and an Al ring (3 cm diameter) served as a cathode. The interelectrode distance was about 8 cm. The electrolyte was cooled using an Unistat Tango chiller (Peter Huber Kältemaschinenbau AG, Offenburg, Germany) and agitated continuously during the anodization. The AAO thickness was controlled coulometrically using a thickness-to-charge density ratio of 0.5 µm cm^2^ C^−1^ [11].

The evolution of pore ordering starting from nucleation on the patterned surface of Al single crystals with subsequent formation of thick AAO was monitored using a step-by-step procedure. First, pre-patterned aluminum was anodized in 0.3 M oxalic acid at 41 V and an electrolyte temperature of 5 °C. Subsequently, selective etching of the obtained AAO in a solution containing 0.2 M CrO_3_ and 0.6 M H_3_PO_4_ at 70 °C was carried out. The resulting Al replica of the bottom side of the AAO surface was characterized using SEM, followed by statistical analysis of the resulting images. All of the above steps were repeated several times. The thickness of AAO grown during a single anodizing step was restricted to 50 µm to prevent diffusion limitations in the AAO channels [10].

The characterization of aluminum crystallographic orientation with subsequent Ga FIB patterning was implemented using a NVision 40 dual beam microscope (Carl Zeiss AG, Oberkochen, Germany) equipped with a Nordlys IIS EBSD detector (Oxford Instruments, Abingdon, UK). A 30 kV Ga FIB with a beam current of 80 pA was used, with a dwell time per concave value equal to 11 ms. The electron backscattered patterns were analyzed using HKL Channel 5 software (Oxford Instruments, Abingdon, UK).

The degree of pore ordering was determined by SEM analysis of the aluminum replicas after the selective dissolution of AAO. SEM images acquisition was performed using Supra 50 VP and Supra 40 instruments (Carl Zeiss AG, Oberkochen, Germany). Statistical analysis of the SEM images was subsequently carried out using the ImageJ program [43] and lab-developed software [44] to reveal the following numerical parameters: the full width at half-maximum (FWHM) of interpore distance distribution (Δ*d*), the fraction of pores with hexagonal coordination (ψ), and the FWHM of the azimuthal orientation distribution of hexagons formed by the six nearest neighbors relative to the central pore (i.e., mosaicity) (φ). To calculate the azimuthal orientation of the considered hexagon, the angles between the lines connecting the central pore with each of its six nearest neighbors and the reference horizontal direction were determined. These angles were then reduced into a basic angle interval of [0°, 60°] by adding or subtracting multiples of 60°. The average of the six reduced angles represented the azimuthal orientation of the hexagon.

## 3. Results and Discussion

**Figure 1:**
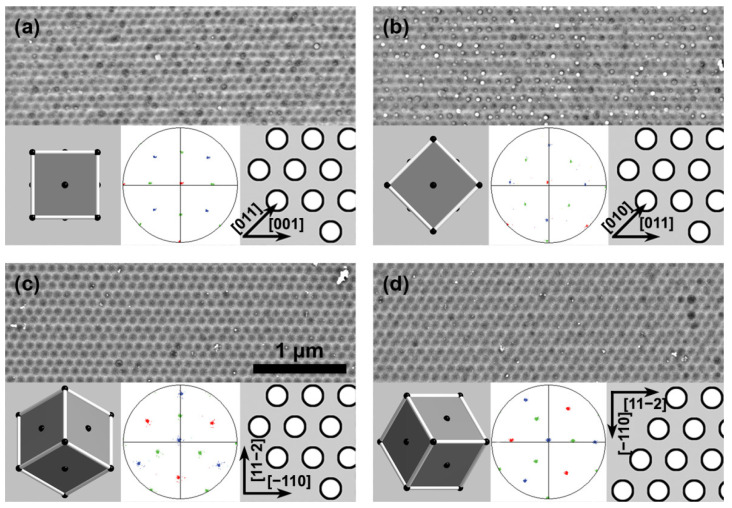
Scanning electron microscopy (SEM) images of the patterned surfaces of Al single crystals: (**a**,**b**) Al(100) and (**c**,**d**) Al(111). The scale bar is the same for all images. The insets in each panel illustrate the schemes of Al unit cell orientation (left), electron backscatter diffraction (EBSD) pole figures (middle), and the orientation relationships between the hexagonal array of concaves and the basis vectors of the Al unit cell (right). Reflections in the EBSD pole figures from various crystallographic planes are shown in different colors: {100} (red), {110} (green), and {111} (blue).

Combining the EBSD and FIB techniques allows the azimuthal orientation of a patterned defect-free array of concaves in the Al surface, which serve as pore nucleation centers, to be matched with the target directions in the Al unit cell (Figure 1). In the case of the Al(100) substrate, two arrays of concaves with a lateral size of 50 × 40 µm^2^ (each containing around 2 × 10^5^ concaves), whose azimuthal orientations differed by an angle of 45°, were formed. One of these represents rows of concaves located along the [001] direction of the Al unit cell (Figure 1a). In the second array, the rows of concaves are placed along the [011] direction (Figure 1b). In the case of the Al(111) substrate (Figure 1c,d), two arrays of the same lateral size containing rows of pore nuclei located along the [−110] and [11–2] directions were formed. There is a 30° azimuthal orientation difference in these arrays.

**Figure 2:**
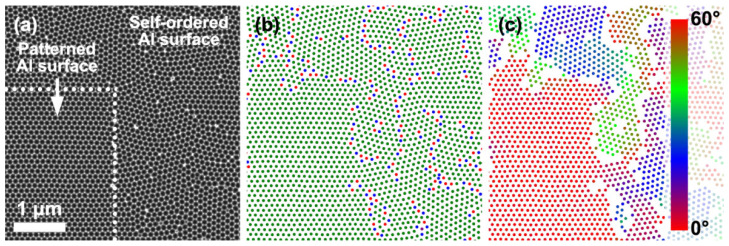
Color coding of SEM data. (**a**) SEM image and (**b**,**c**) corresponding color-coded maps, where the colors indicate the following: (**b**) the number of the nearest neighbors (five—red, six—green, and seven—blue) and (**c**) azimuthal orientation of hexagons formed by the nearest neighbors of the considered pore reduced into a basic angle interval of [0°, 60°]. The horizontal direction is used as a reference azimuthal direction.

To perform a quantitative analysis of the porous structures formed during anodization of the patterned substrates, SEM images of the Al surface after the selective dissolution of AAO were color-coded (Figure 2a–c). The color map in Figure 2b visualizes the number of the nearest neighbors around the considered pore. Pore positions with hexagonal coordination are marked in green, whereas pores near defects possessing five and seven nearest neighbors are colored red and blue, respectively. As shown, the patterned area does not have any defects, whereas the Al replica of the self-ordered AAO consists of domains—areas with a hexagonal ordering—and has numerous pore positions with five and seven nearest neighbors at the domain boundaries. For all pores with hexagonal coordination, the azimuthal orientation of the hexagon formed by their nearest neighbors was calculated (Figure 2c). The azimuthal orientation is constant within the patterned area, whereas domains are misaligned from one other in the self-ordered area. A low-magnification SEM image of the Al surface after the selective dissolution of AAO formed on the FIB-patterned surface is shown in Supplementary Materials, Figure S1. The corresponding color maps illustrate the prevalent hexagonal arrangement of the pores and narrow distribution of the target azimuthal orientations (Supplementary Materials, Figure S1b,c).

The thickness-dependent azimuthal orientation of AAO, formed on a patterned Al(111) single crystal, is presented as a series of color-coded maps in Supplementary Materials, Figure S2. Only the pores in hexagonal coordination are color-coded, whereas the pores in faulty coordination are marked in white. Visual comparison of the color maps indicates a progressive disordering of the porous structure with increasing total AAO thickness. In the case of FIB patterning along the [11–2] direction, the ‘white’ zones consisting of pores without apparent hexagonal coordination and the ‘blue’ zones with 30° misorientation from the initial patterned rows of concaves gradually appear and increase in size for thicker AAO cases. In the case of FIB patterning along the [−110] direction, deviation from the artificially created defect-free array of concaves is also seen, however, the array as a whole is more uniform.

**Figure 3:**
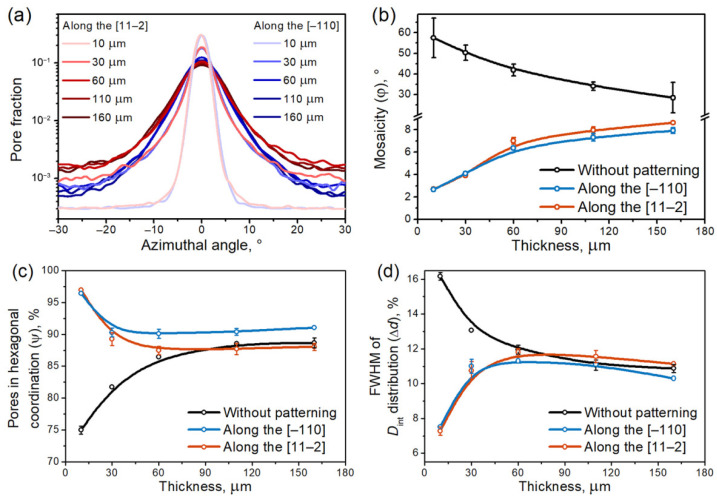
Thickness-dependent degree of pore ordering in anodic aluminum oxide (AAO) formed by anodization of pre-patterned Al(111) single crystals. (**a**) Azimuthal orientation distributions of hexagons formed by the six nearest neighbors of the considered pore, (**b**) the mosaicity of the porous structure, (**c**) the fraction of pores in hexagonal coordination, and (**d**) the FWHM of interpore distance (*D*_int_) distribution. The error bars correspond to the standard deviation values calculated from at least four SEM images each containing about 5 × 10^4^ pores. The B-spline curves in (**b**–**d**) are given to guide the eye.

Color maps can also be used to represent the distributions of the hexagons’ azimuthal orientations (Figure 3a). The narrow width of the lightest red and blue curves corresponds to the low azimuthal misorientation of porous structure in the case of 10-µm-thick AAO. The azimuthal profiles for Al replicas become broader with increasing AAO thickness. Distinctive color nonuniformity at AAO thickness values above 100 µm in the case of FIB patterning along the [11–2] direction results in high background values in comparison with the case of FIB patterning along the [−110] direction (see red and blue curves respectively in Figure 3a). The azimuthal distributions were quantified by fitting the experimental data with a Gaussian function. The FWHM of the fitting function represents the mosaicity of the AAO structure. The resulting thickness dependencies in the mosaicity (Figure 3b) demonstrate smooth growth that is in good agreement with the increase in imperfections observed in the color maps (Figure S2). In the case of 150-µm-thick AAO, the mosaicity of the porous structure formed on the patterned Al is about three times smaller than that of the self-ordered porous structure (black curve in Figure 3b).

The fraction of pores in hexagonal coordination (Figure 3c) and the FWHM of *D*_int_ distribution (Figure 3d) demonstrate nearly the same behavior: a maximum degree of ordering (i.e., low number of point defects) was revealed at small AAO thicknesses, with a subsequent decrease and stabilization during the growth of AAO. In particular, ψ changes insignificantly for AAO thicknesses from 50 to 150 µm, reaching values of around 90%. The value of Δ*d* is about 11% in the same thickness interval. We stress that the self-ordered porous structure grown on the Al(111) surface without patterning exhibits similar ψ and Δ*d* values at an AAO thickness of 100 µm. Nevertheless, a crystallography impact becomes evident for porous films thicker than 50 µm; the AAO grown on the Al with rows of concaves oriented along the [11–2] direction reveals a lower fraction of pores in hexagonal coordination and a higher *D*_int_ dispersion compared to AAO formed on Al(111) with concaves patterned along the [−110] direction.

Anodization of pre-patterned Al(100) leads to lower-defect pore arrays (Supplementary Materials, Figure S3), as demonstrated by the high color uniformity and sparse white-marked point defects, compared with the Al(111) case. The evaluation of two arrays with 45° azimuthal misorientation reveals a high density of point defects at small AAO thicknesses in the case of FIB patterning along the [011] direction. In contrast, in the case of [001]-direction patterning, color nonuniformity appears at high AAO thickness values, proving the formation of domains with different azimuthal orientations.

**Figure 4:**
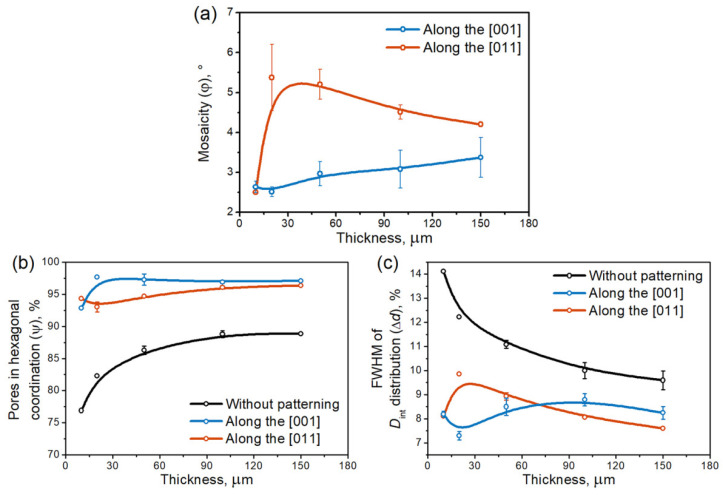
Thickness-dependent degree of pore ordering in AAO formed by anodization of pre-patterned Al(100) single crystal: (**a**) the mosaicity of porous structure, (**b**) the fraction of pores in hexagonal coordination, and (**c**) the FWHM of *D*_int_ distribution. The error bars correspond to the standard deviation calculated from at least four SEM images, each containing around 5 × 10^4^ pores. The B-spline curves are given to guide the eye.

Figure 4 illustrates the thickness-dependent degree of pore ordering in AAO formed on the patterned and flat surfaces of Al(100) single crystals. Two opposite trends in the evolution of mosaicity can be identified (Figure 4a). In the case of concave rows located along the [011] direction, a decrease in φ value is observed due to co-direction between the patterned rows of concaves and the preferred azimuthal orientation of the AAO structure relative to the Al unit cell (red curve in Figure 4a). However, an opposite increase in the φ-value for AAO formed on patterned Al with concave rows located along the [001] direction is observed (blue curve in Figure 4a). The disordering of the porous structure is caused by a high misorientation angle between the patterned rows of concaves and the preferred azimuthal orientation of the AAO structure from a crystallographic point of view. Notably, the self-ordered AAO grown on the Al(100) substrate is characterized by the absence of a preferred azimuthal orientation [19] and, thus, the φ-values for self-ordered AAO are not shown in Figure 4a.

The same opposing trends are observed for the fraction of pores in hexagonal coordination (Figure 4b) and the FWHM of *D*_int_ distribution (Figure 4c), i.e., the increase in the degree of pore ordering for nucleation centers oriented along the [011] direction and the increase in structural imperfection in the case of concave rows located along the [001] direction. We highlight that in both cases, the ψ value is about 95% and changes negligibly with increasing AAO thickness. Such an ordering degree is unattainable for self-ordered AAO, where ψ is below 87% even after long-term anodization. Probably, the observed difference in ordering parameters of small thicknesses of AAO formed using two patterned arrays with a 45° misorientation angle is caused by variance in the quality of as-prepared concaves (e.g., in their depth or diameter) and will be a subject of future study.

In summary, the observed trends are in good agreement with the recently proposed origin of the long-range arrangement of AAO porous structures [37]. The preferred azimuthal orientation of self-ordered hexagonal lattices in the case of anodization of Al(100) and Al(111) coincides with the <110> directions in the Al unit cell. These directions are known for the highest atomic packing density in fcc crystals and act as bearing axes for the alignment of pore rows in the plane of AAO films [27,37]. In the case of patterned Al substrates, azimuthal misorientation between artificially created concave rows and the <110> direction in the Al unit cell induces progressive degradation of the pore arrangement with increasing AAO thickness.

## 4. Conclusions

In this study, the relationship between the pattern of pore nuclei and the crystallographic orientation of aluminum in pore ordering process was elucidated. Defect-free pore nuclei arrays with different azimuthal orientations relative to the basis vectors of the Al unit cell were prepared using FIB patterning of Al(100) and Al(111) single-crystal substrates. Subsequent step-by-step anodization in 0.3 M oxalic acid at 41 V with an electrolyte temperature of 5 °C allowed us to quantify the dependence of the pore ordering degree on the thickness of AAO structures. The study results prove the existence of a preferred azimuthal alignment of the pore nuclei array relative to the basis vectors of the Al unit cell, which is favorable for the creation of AAO with a high degree of ordering. In particular, the <110> directions are advised for anchoring during pre-patterning of the Al surface before anodization. In the case of an unspecified azimuthal orientation of the pore nuclei array, crystallography-affected disordering of the AAO structure with increasing film thickness may occur, although the resulting crystallographic impact is relatively small. Our findings have important implications for the preparation of defect-free porous films over 100 µm in thickness that are crucial for a variety of AAO applications, e.g., creating 2D/3D photonic crystals and metamaterials. The approach based on the FIB patterning of metal surface along a distinct crystallographic orientation potentially can be applied for preparing porous anodic oxide films with highly ordered structure on titanium and other valve metals.

## Data Availability

The data presented in this study are available on request from the corresponding author.

## Supplementary Materials

The following supporting information can be downloaded at: https://www.mdpi.com/article/10.3390/nano12091417/s1, Figure S1: Example of statistical analysis of the low-magnification SEM image; Figure S2: Thickness evolution of the color maps during anodization of the pre-patterned Al(111) substrate; Figure S3: Thickness evolution of the color maps during anodization of the pre-patterned Al(100) substrate.
