# Supplementary material for: Pore Ordering in Anodic Aluminum Oxide: Interplay between the Pattern of Pore Nuclei and the Crystallographic Orientation of Aluminum
Source: Nanomaterials (Basel). 2022 Apr 20;12(9):1417. doi: 10.3390/nano12091417 (PMC9104029; doi:10.3390/nano12091417)
Supplement: Supplementary file 1 [file nanomaterials-12-01417-s001.zip › nanomaterials-1683161-supplementary.pdf]

nanomaterials

SUPPLEMENTARY MATERIALS

Pore Ordering in Anodic Aluminum Oxide: Interplay between the Pattern of Pore Nuclei and the Crystallographic Orientation of Aluminum

Ilya V. Roslyakov [1,2], Stepan V. Sotnichuk [1,3], Sergey E. Kushnir [1,4], Lev A. Trusov [4,5], Ivan V. Bozhev [6,7] and Kirill S. Napolskii [1,4,*]

[1] Department of Materials Science, Lomonosov Moscow State University, 119991 Moscow, Russia; ilya.roslyakov@gmail.com (I.V.R.); sotnya777@mail.ru (S.V.S.); kushnir@elch.chem.msu.ru (S.E.K.)
[2] Kurnakov Institute of General and Inorganic Chemistry RAS, 119991 Moscow, Russia
[3] Dukhov Research Institute of Automatics (VNIIA), 127055 Moscow, Russia
[4] Department of Chemistry, Lomonosov Moscow State University, 119991 Moscow, Russia; trusov@inorg.chem.msu.ru
[5] Department of Materials Science, MSU-BIT University, Shenzhen 517182, China
[6] MSU Quantum Technology Centre, 119991 Moscow, Russia; bozhjev.ivan@physics.msu.ru
[7] Department of Physics, Lomonosov Moscow State University, 119991 Moscow, Russia
* Correspondence: kirill@inorg.chem.msu.ru

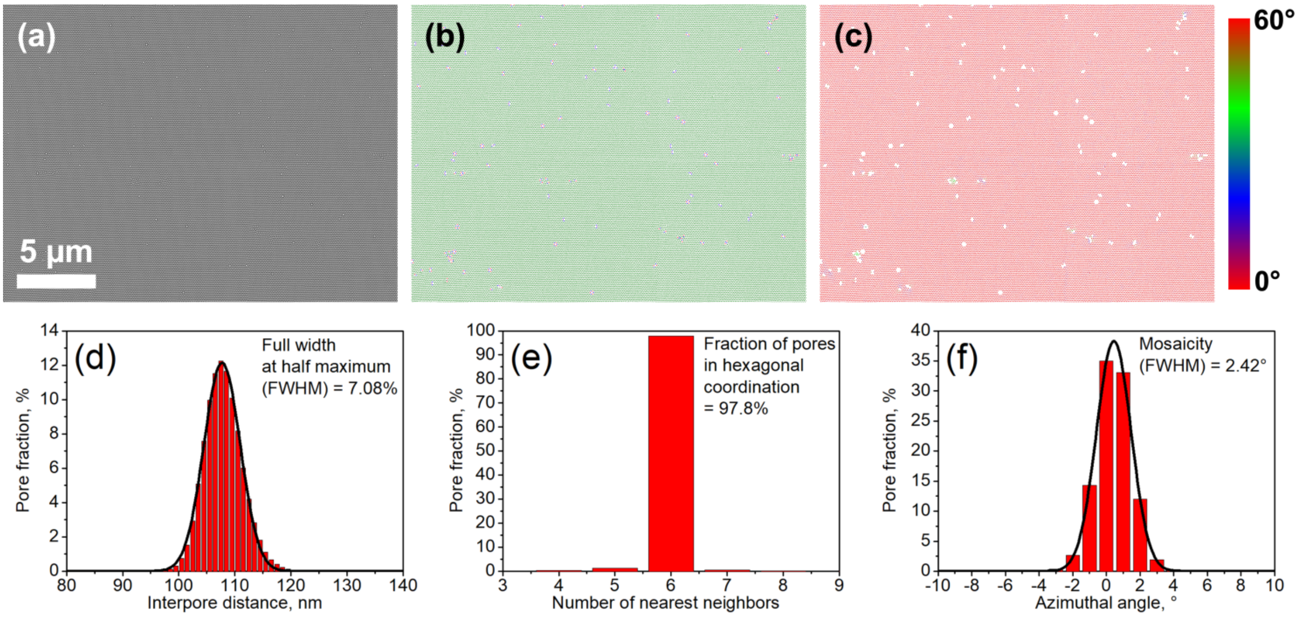

Figure S1. Low-magnification scanning electron microscopy (SEM) image of Al surface after selective dissolution of porous anodic aluminum oxide (AAO) (**a**) and corresponding color-coded maps, where the colors indicate the following: (**b**) the number of the nearest neighbors (five — red, six — green, and seven — blue); (**c**) azimuthal orientation of hexagons formed by the nearest neighbors of the considered pore reduced into a basic angle interval of [0°, 60°]. The horizontal direction is used as a reference azimuthal direction. The SEM image contains of about 5×10^4 pores. The bottom row of panels illustrates the following: (**d**) distributions of interpore distance; (**e**) the number of the nearest neighbors; (**f**) azimuthal orientation distributions of hexagons. The solid lines represent the best fits of the experimental data by a Gaussian function.

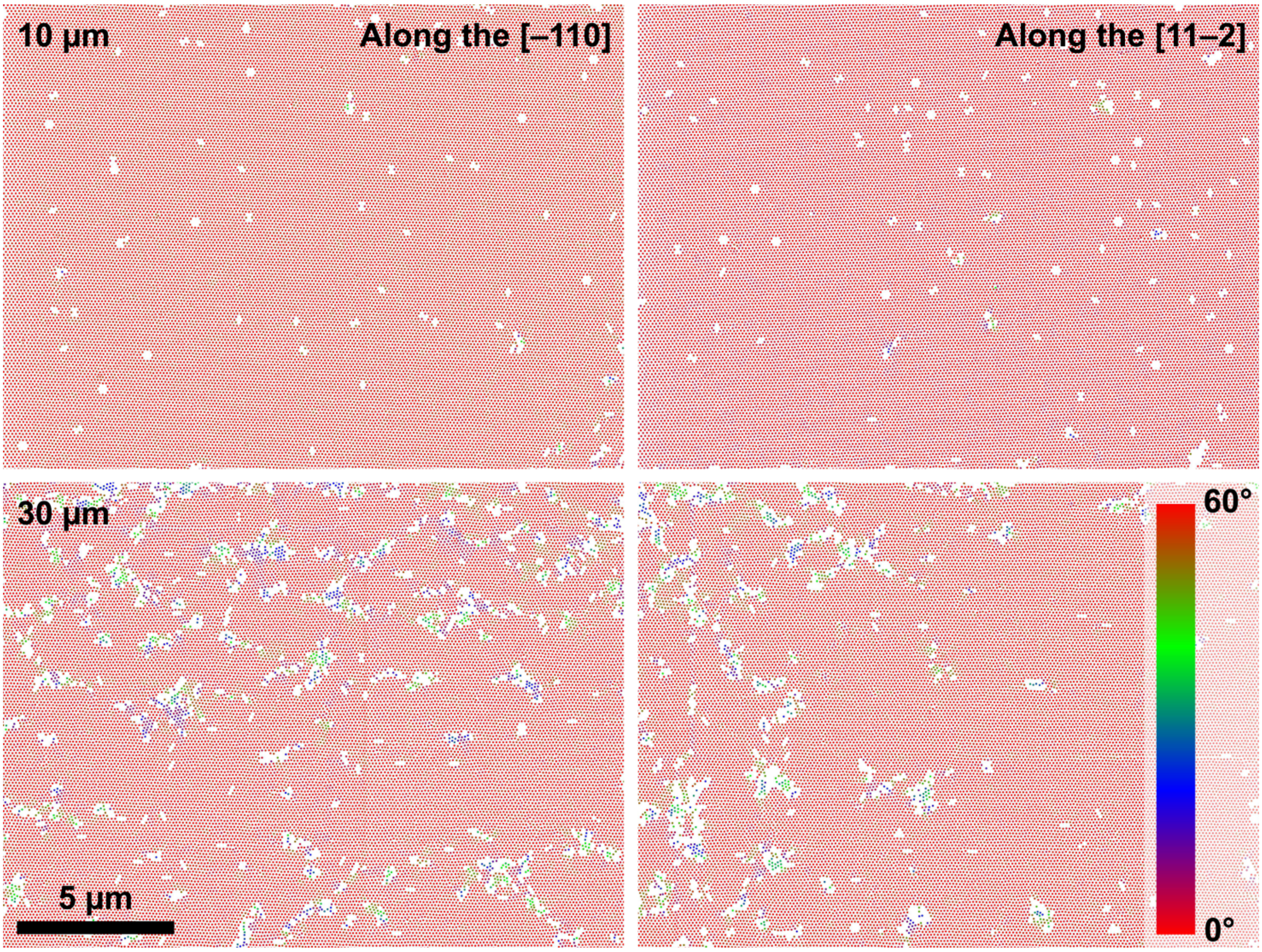
10 μm
Along the [–110]
Along the [11–2]
30 μm
5 μm
60°
0°

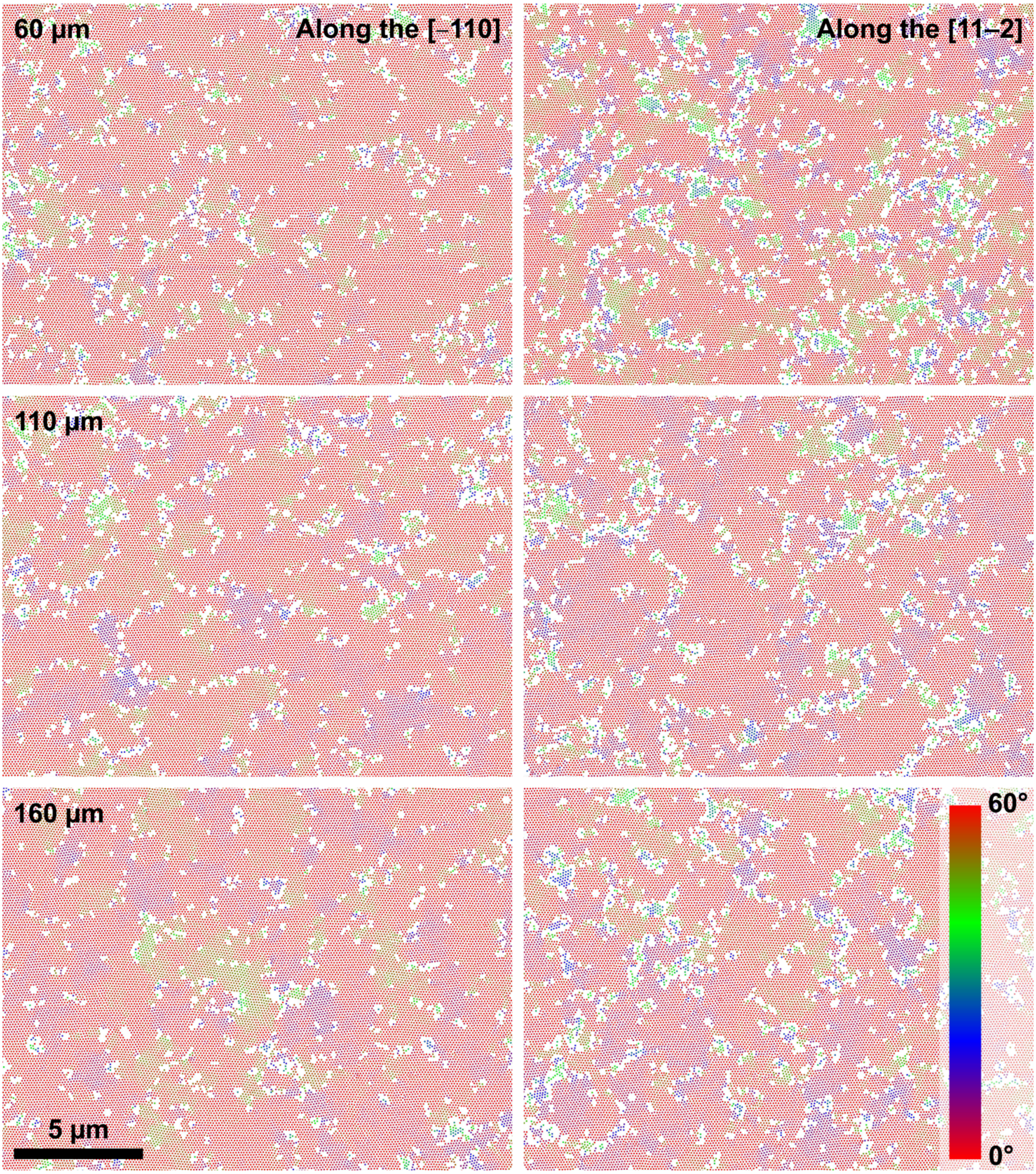

Figure S2. Thickness evolution of the porous structure of anodic aluminum oxide during anodization of the pre-patterned Al(111) single crystal in 0.3 M oxalic acid at 41 V and electrolyte temperature of 5 °C. The pores are color-coded in accordance with the in-plane azimuthal orientation of the hexagon formed by the six nearest neighbors of the considered pore. The horizontal direction is used as a reference azimuthal direction. Rows of pore nuclei were formed by focus ion beam etching along the [–110] direction (left column, corresponds to Figure 1c in the main text) and along the [11–2] direction (right column, corresponds to Figure 1d in the main text) of Al unit cell. Scale bar and color scale are the same for all maps.

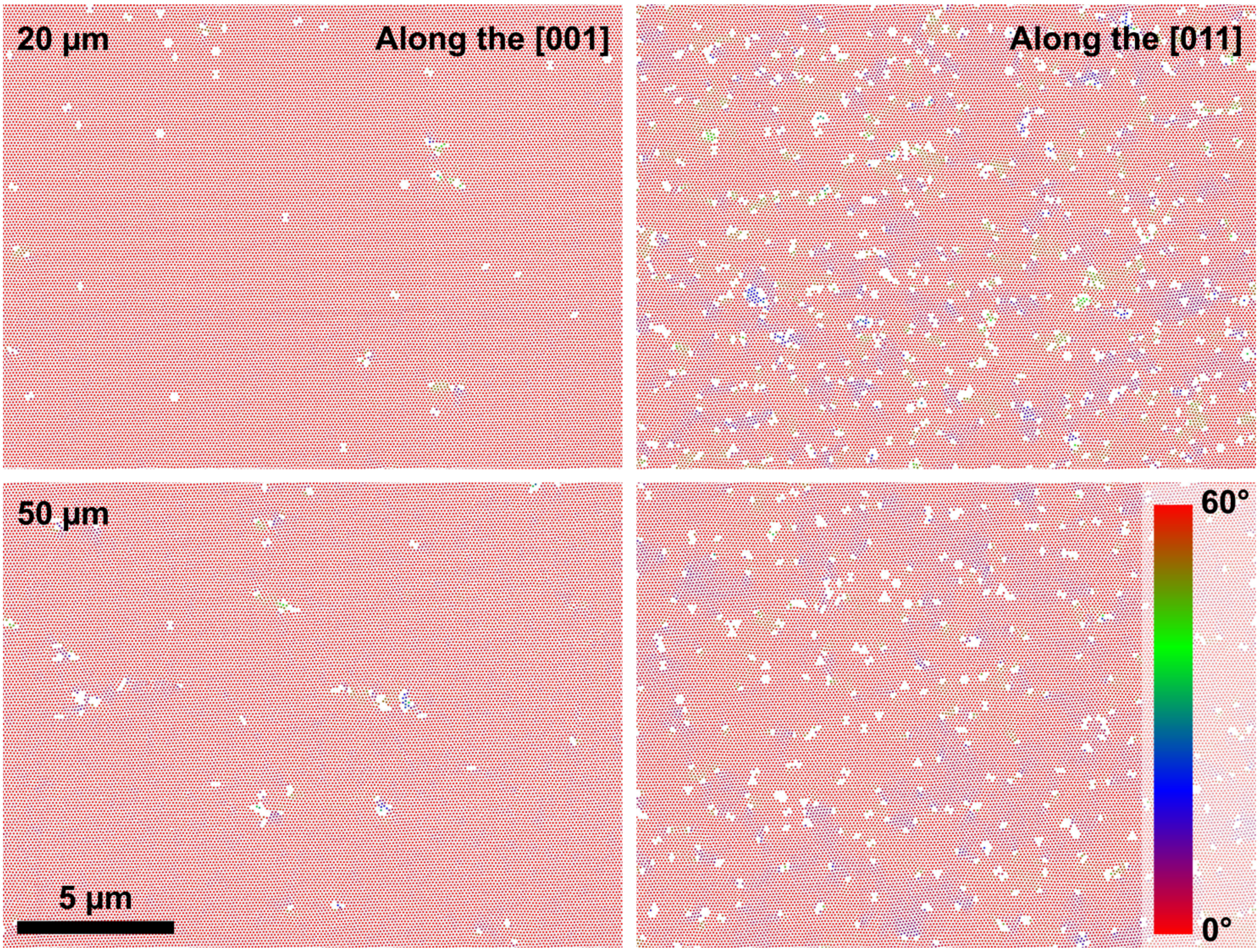
20 μm
Along the [001]
Along the [011]
50 μm
5 μm
60°
0°

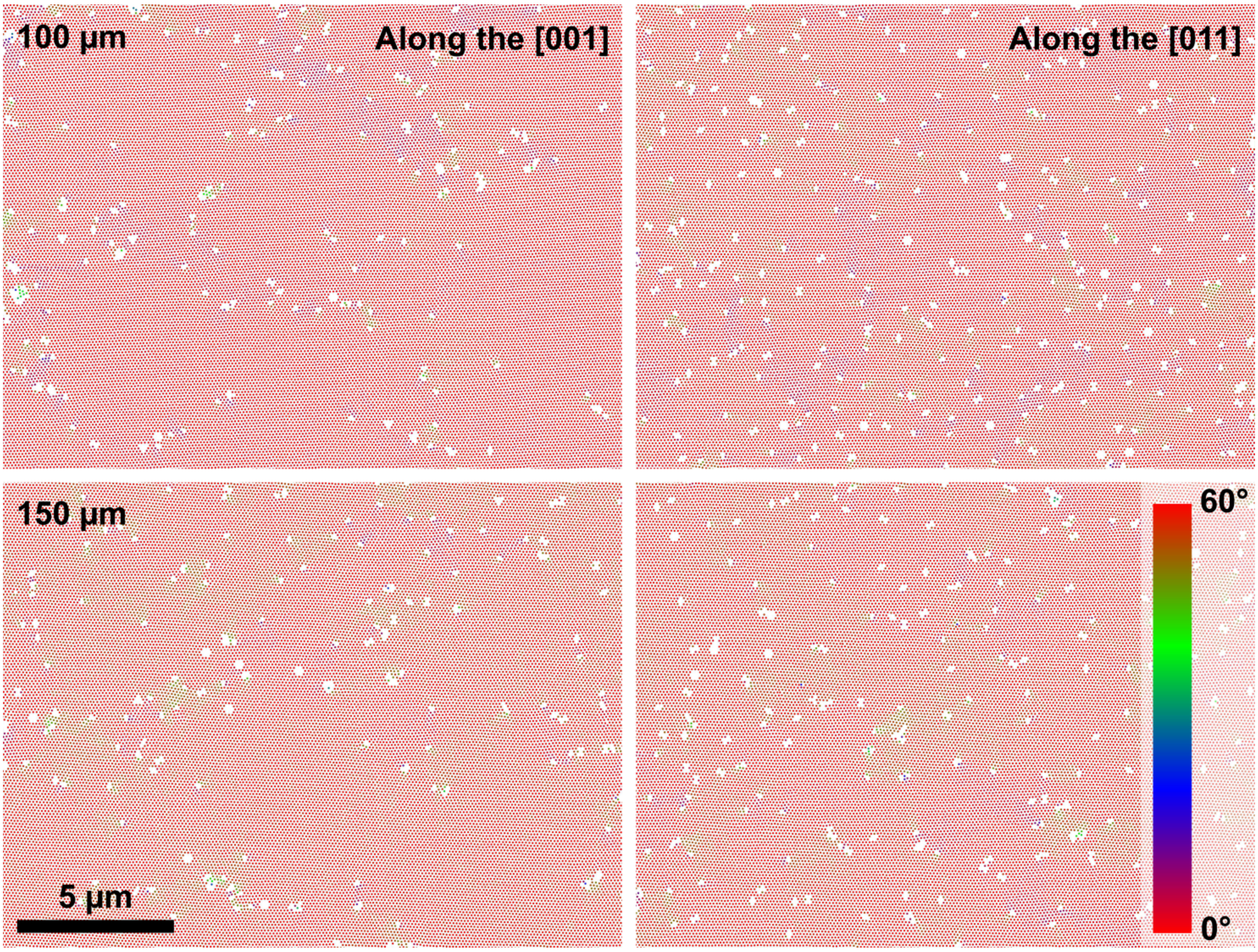

Figure S3. Thickness evolution of the porous structure of anodic aluminum oxide during anodization of the pre-patterned Al(100) single crystal in 0.3 M oxalic acid at 41 V and electrolyte temperature of 5 °C. The pores are color-coded in accordance with the in-plane azimuthal orientation of the hexagon formed by the six nearest neighbors of the considered pore. The horizontal direction is used as a reference azimuthal direction. Rows of pore nuclei were formed by focus ion beam etching along the [001] direction (left column, corresponds to Figure 1a in the main text) and along the [011] direction (right column, corresponds to Figure 1b in the main text) of Al unit cell. Scale bar and color scale are the same for all maps.
